# Screening of Mutants Related to the C_4_ Photosynthetic Kranz Structure in Foxtail Millet

**DOI:** 10.3389/fpls.2018.01650

**Published:** 2018-11-14

**Authors:** Mingzhao Luo, Shuo Zhang, Chanjuan Tang, Guanqing Jia, Sha Tang, Hui Zhi, Xianmin Diao

**Affiliations:** Institute of Crop Sciences, Chinese Academy of Agricultural Sciences, Beijing, China

**Keywords:** foxtail millet, C_4_ photosynthesis, Kranz structure, EMS mutant, leaf mutant

## Abstract

C_4_ plants exhibit significantly higher photosynthetic, water and nutrient use efficiency compared with C_3_ plants. Kranz anatomy is associated with many C_4_ plants in which bundle sheath cells surround the veins and are themselves surrounded by mesophyll cells. This specialized Kranz anatomy is elucidated as an important contributor to C_4_ photosynthetic activities in C_4_ plant. Characterizing the molecular basis of Kranz structure formation has become a key objective for studies of C_4_ photosynthesis. However, severe mutants that specifically disrupt Kranz anatomy have not been identified. In this study, we detected 549 stable ethyl methane sulfonate-induced foxtail millet (cultivar Yugu1) mutants related to leaf development and photosynthesis among 2,709 mutants screened (M_3_/M_4_ generation). The identified mutants included 52 that had abnormal leaf veins (with abnormal starch accumulation based on iodine staining). Each of the 52 mutants was characterized through an analysis of leaf morphology, and through microscopic observations of leaf tissue sections embedded in resin and paraffin. In total, 14 mutants were identified with abnormal Kranz structures exemplified by small bundle sheath cell size. Additional phenotypes of the mutants included poorly differentiated mesophyll and bundle sheath cells, increased vein density and the absence of chloroplasts in the bundle sheath cells. Kranz structure mutations were accompanied by varying leaf thickness, implying these mutations induced complex effects. We identified mutations related to Kranz structure development in this trial, which may be useful for the mapping and cloning of genes responsible for mediating Kranz structure development.

## Introduction

Photosynthesis, the driver for life on this planet, includes many carbon fixation pathways (e.g., C_3_, C_4_, and crassulacean acid metabolism). In all photosynthetic pathways, a bi-functional enzyme-ribulose-1,5-bisphosphate carboxylase/oxygenase (Rubisco) is needed to fix CO_2_ into carbohydrates. The C_4_ photosynthetic pathway completes the initial and secondary CO_2_ fixations using two distinct photosynthetic cell types, which form a distinct CO_2_-concentrating mechanism that significantly improves the carboxylation efficiency of Rubisco. Consequently, C_4_ plants exhibit a higher photosynthetic efficiency, biomass production, and water and nutrient usage than C_3_ plants under hot, dry conditions that favor stomatal closure (Diao et al., 2014; Saha and Blumwald, 2016). These characteristics enhance the ability of plants to grow in increasingly arid environments and decrease the need for fertilizer applications. However, many agriculturally important crops, such as rice, wheat, soybean, and potato, are C_3_ plants. Thus, the suggestion that introducing the C_4_ pathway into C_3_ plants may improve productivity (Zhu et al., 2010; Li et al., 2011; Rizal et al., 2012; Wang et al., 2017) prompted the initiation of The C_4_ Rice Project^1^ by a group that included leading rice researchers. Promising results have included the successful transfer of genes encoding key C_4_ pathway enzymes into C_3_ plants to improve photosynthetic efficiency (Ku et al., 1999; Chi et al., 2004). However, researchers have been unable to generate C_3_ plants with a highly efficient CO_2_-concentrating mechanism even after transferring one or more genes encoding C_4_ cycle metabolic enzymes (Westhoff and Gowik, 2010). This inability has been partly due to a lack of a leaf Kranz structure, which is important for the spatial separation of enzymatic activities related to carbon fixation (Kromdijk et al., 2014).

All grass family C_4_ crops have a typical Kranz structure (Christin et al., 2013) consisting of an inner layer of relatively large bundle sheath cells around a vascular bundle. The bundle sheath cells are surrounded by a layer of mesophyll cells that are compactly arranged to form a concentric ring-shaped structure. These are generally only two mesophyll cells between vascular bundles in C_4_ leaves as opposed to up to 18 cells in C_3_ leaves (Sage et al., 2014). Elucidating the regulatory mechanisms underlying the development of Kranz structure has been the focus of research into C_4_ photosynthesis (Langdale, 2011). Some researchers have examined genes differentially expressed between C_3_ and C_4_ plants (Wang et al., 2014) or tissues (Wang et al., 2013). Other studies of C_4_ plants have applied genome and transcriptome sequencing techniques to analyze bundle sheath and mesophyll cells (Li J. et al., 2010; Covshoff et al., 2013) at different developmental stages (Li P. et al., 2010). These studies identified several differentially expressed genes between leaf development stages before and after the formation of the Kranz structure, and between mesophyll and bundle sheath cells. Although these studies have suggested several candidate genes, no functional tests of these candidates have revealed regulators of the process.

Geological studies (e.g., involving carbon isotopes) and phylogenetic analyses of various species have suggested that C_4_ plants evolved from C_3_ plants, with structural and gene expression changes having occurred over time. More than 66 independent events took place that caused some C_3_ plants to evolve into C_4_ plants, which is considered as the best example of convergent evolution (Hibberd, 2002; Sage et al., 2011; Rizal et al., 2012; Christin and Osborne, 2013). The first stage of this evolution involved structural changes, including the development of a higher leaf vein density, larger bundle sheath cell volume, as well as increased abundance and altered localization of organelles (e.g., chloroplasts and mitochondria) in bundle sheath cells. Additionally, genes encoding specific enzymes (e.g., PEPC and GDC) began to show cell-type specific enrichment. Moreover, CO_2_ fixation occurred first in the outer leaf cells, after which the carbohydrates derived from CO_2_ were fixed in the inner bundle sheath cells (Mckown and Dengler, 2007, 2009; Sage and Zhu, 2011). Studies revealed that some positive/negative regulators in the developing endoderm might have been important for the formation of C_4_ structures (Slewinski et al., 2012). Researchers have mined for genes related to the development of C_4_ structures by selecting and identifying mutants, including maize mutants with abnormal leaf veins (Slewinski et al., 2012), rice mutants with increased leaf vein density (Feldman et al., 2014), and sorghum mutants with decreased leaf vein density (Rizal et al., 2015). Several transcription factors that help regulate BSC differentiation in C_4_ species have been identified, including SCR and SHR (Gardiner et al., 2011; Slewinski et al., 2012).

Maize and sorghum have been important model organisms in studies of C_4_ plants, but they are large plant species with complex genomes. In contrast, green foxtail and foxtail millet are small in stature and have simpler genomes. Foxtail millet originated in the Yellow River basin of China, and represents a domesticated form of green foxtail. The self-pollinating diploid foxtail millet exhibits characteristics that make it useful for research, including a high seed set per spike, compact size, simple growth condition requirements, relatively small genome (490 Mb), and a high transformation efficiency. Therefore, this plant species is gradually being used as a new model organism for studies of the C_4_ photosynthetic mechanism (Doust et al., 2009; Diao et al., 2014; Saha and Blumwald, 2016). Mutant lines exhibiting abnormal development of C_4_ photosynthetic structures may be very important for identifying and cloning of responsible genes. In this study, genetically stable ethyl methanesulfonate (EMS) mutants generated using the Yugu1 cultivar were analyzed for screening of changes in leaf appearance and vein density, sugar accumulation, and Kranz structural features. Mutants with abnormal leaf veins and Kranz structures were identified, and may be useful for elucidating the molecular mechanism underlying Kranz structure and development.

## Materials and Methods

### Tested Materials

We previously developed EMS-based methods for mutagenizing foxtail millet (Li W. et al., 2010) to establish a stable mutant library for foxtail millet cultivar Yugu1. A total of 2,709 M_3_/M_4_-generation mutants with stable phenotypes were preliminarily screened. Wild-type Yugu1 (maintained in our laboratory through many generations of self-pollinations) was used as the C_4_ control plant, while the wild-type Nipponbare rice cultivar (provided by Xingguo Ye from the Institute of Crop Sciences, Chinese Academy of Agricultural Sciences) was used as the C_3_ control plant.

### Growth Conditions

Plants were grown in a 3 m × 0.5 m plot using standard agronomic practices (e.g., irrigation, weeding, and pesticide spraying) at the Institute of Crop Sciences, Chinese Academy of Agricultural Sciences in Shunyi district, Beijing, China.

### Fixation of Materials

For each mutant line, we selected 6–8 similarly growing and normally developing plants that were free of diseases and insect pests. The middle parts of completely unfolded leaves were collected at the 8-leaf stage. The FAA-fixed samples (about 0.5 cm × 1 cm) were placed in a vacuum for more than 0.5 h. Samples were then re-fixed in new FAA solution for 24 h, washed three times with 70% ethanol, and then preserved. The glutaraldehyde-fixed samples (about 1 mm × 2 mm) were placed in a vacuum for 3–4 h, after which they were fixed again in new glutaraldehyde solution.

### Microscopic Observation

The Anyty V500IR/UV portable digital microscope (3R Eddytek Corp., Beijing, China) was used to observe and photograph the leaf veins of striped mutants, three individuals for each mutant line were sampled for observation.

### Iodine Staining

Fixed samples preserved in 70% ethanol were dehydrated with increasing ethanol concentrations (85%, 95%, and 100%). They were then treated with different ethanol:xylene mixtures (2:1, 1:1, 1:2, and pure xylene). Samples were gradually exposed to absolute ethanol (ethanol:xylene mixtures, 1:2, 1:1, 2:1, and pure ethanol; kept in each concentration for 1 h), and then stained with I_2_-KI for 12 h. Changes in leaf vein density and starch content were then observed microscopically. Image J^2^ was used to calculate vein density and carbohydrate accumulation in mutant individuals.

### Preparation of Paraffin Sections

Leaf cross-sections were further observed using paraffin sections, which were prepared using a modified version of a traditional method. Samples preserved in 70% ethanol were dehydrated in 85%, 90%, and 100% (twice) ethanol, and then treated with different ethanol:xylene mixtures [2:1, 1:1, 1:2, and pure xylene (twice)]. Each treatment lasted 40–60 min, with the duration determined based on the growth condition of mutants. Samples were then embedded in paraffin, and 10-μm sections were generated using the Leica RM2250 microtome. The sections were placed on distilled water on microscope slides to ensure they were sufficiently spread out before being heated for more than 48 h. The prepared sections were stained with 0.2% toluidine blue and then sealed with neutral balsam. Samples were observed and photographed using a Leica microscope.

### Preparation of Resin Sections

We collected leaves from mutants with an abnormal Kranz structure as observed using the paraffin section. We also selected 6–8 mutant individuals per mutant line, and leaf samples were fixed with 2.5% glutaraldehyde and then rinsed three times (10 min each) with phosphate buffer. Samples were fixed in 1% osmic acid for 1 h, and then rinsed three times with double-distilled H_2_O (10 min each). Samples were gradually dehydrated in different concentrations of ethanol and then incubated in acetone for 10 min. They were incubated overnight in a 1:1 acetone:resin mixture, with mild shaking, after which they were incubated overnight in pure resin before being embedded in resin polymer. Samples were sliced into 500–800 Å sections using an ultramicrotome, and then picked up by copper mesh, placed on microscope slides, and stained with toluidine blue. The sections were observed and photographed using a Leica microscope.

### Reagents

(1) I_2_-KI staining reagent: 1 g I_2_ and 8 g KI were dissolved in distilled water for a final volume of 100 ml. The solution was stored in an amber laboratory bottle.

(2) FAA: 5 ml acetic acid, 5 ml formaldehyde, and 90 ml 70% ethanol were thoroughly mixed.

(3) 2.5% glutaraldehyde fixative: 10 ml 25% glutaraldehyde, 40 ml distilled water, and 0.2 M PBS were thoroughly mixed. The solution was stored at 4°C.

## Results

### Preliminary Screening and Classification of EMS Mutants

A total of 549 mutants from 2,709 stable EMS mutants library was screened based on their phenotypes which show decrease biomass, such as decreased fertility, lax panicle, or leave color variation. We observed that 40.62% of the 549 mutants produced abnormal leaves, among the leaf mutants are list in Table 1.

**Table 1:** Phenotypes of 549 mutants after a preliminary screen.

Phenotypes	Numbers of mutants	Total	Ratio (%)	Phenotypes	Numbers of mutants	Total	Ratio (%)
White striped	48	549	8.74	Yellow leaf	22	549	4.01
Yellow striped	19	549	3.46	Lax panicle	54	549	9.84
Light white striped	39	549	7.10	Erect leaf	67	549	12.20
Yellowish striped	11	549	2.00	Decreased fertility	75	549	13.66
Disease-spot leaf	16	549	2.91	Tight spikelet	99	549	18.03
Abnormal leaf striped and shape	16	549	2.91	Early browning	20	549	3.64
Abnormal leaf shape	52	549	9.47	Late heading	11	549	2.00

**FIGURE 1:**
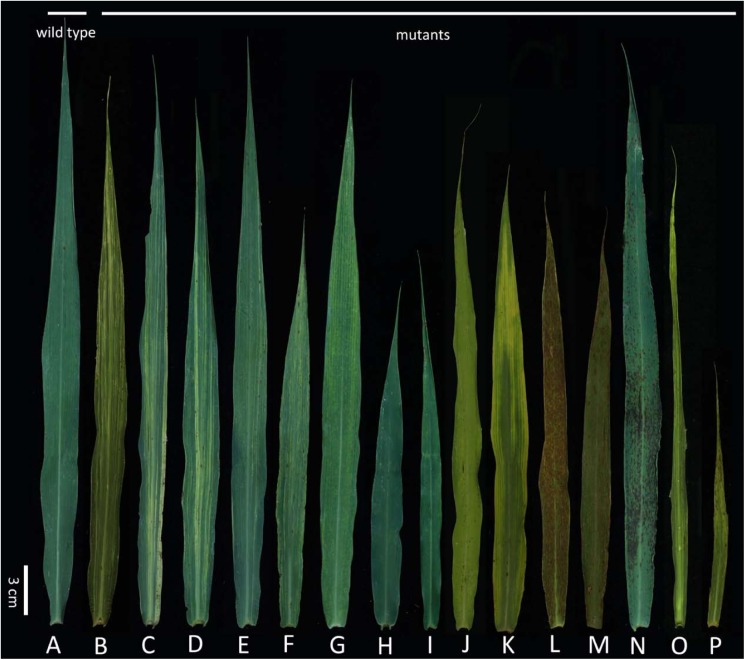
Flag leaf of diverse mutants. **(A)** Wild-type Yugu1; **(B–P)** examples of leaf mutants; **(B)** light white striped; **(C–D)** white striped; **(E)** yellowish striped; **(F)** yellow striped; **(G)** yellowish striped; **(H,I)** abnormal leaf shape; **(J,K)** yellow leaf; **(L-N)** disease-spot leaf; **(O–P)** abnormal leaf striped and shape. Scale: 3 cm.

There were many types of leaf mutants. The wild-type Yugu1 leaves were dark green with a uniformly arrayed leaf vein (Figure 1A). Meanwhile, leaf mutants produced abnormal leaves in terms of striped, shape, and color (Figures 1B–P). Abnormal striped (mainly white and yellow) were the most commonly observed leaf mutations. In some cases (e.g., *t81* and *t104*), white striped were present on the whole leaf (Figures 1B,C). Some mutants (e.g., *t11*) produced leaves with several large white striped (Figure 1D), while other mutants (e.g., *t71*) had normal-sized leaves that were covered with slender white striped (Figure 1E). In some cases (e.g., *t6*), mutants grew thin flag leaves with yellow striped (Figure 1F). Other mutants (e.g., *t53*) had thin yellow striped at the leaf tip, but the rest of the leaf was normal (Figure 1G). Some mutants produced leaves with abnormal sizes and shapes, including *t17*, which had relatively small leaves (Figure 1H) and *t57*, which had narrow leaves (Figure 1I). In mutants with abnormally colored leaves, some produced completely yellow leaves (e.g., *t124*; Figure 1J), while others had leaves that were yellow only at the tip (e.g., *t72*; Figure 1K). Many phenotypes were observed for mutants with lesions. For example, severe brick-red rust was detected on all leaves of some mutants (e.g., *t121*; Figure 1L), while other mutants had small punctum spots (e.g., *t33*; Figure 1M) or black rust spots (e.g., *t35*; Figure 1N) on the leaves. Some mutants had abnormally shaped and striped leaves [e.g., *t14* (Figure 1O) and *t68* (Figure 1P)].

### Microscopic Analysis of the Leaf Veins of Striped Mutants

**FIGURE 2:**
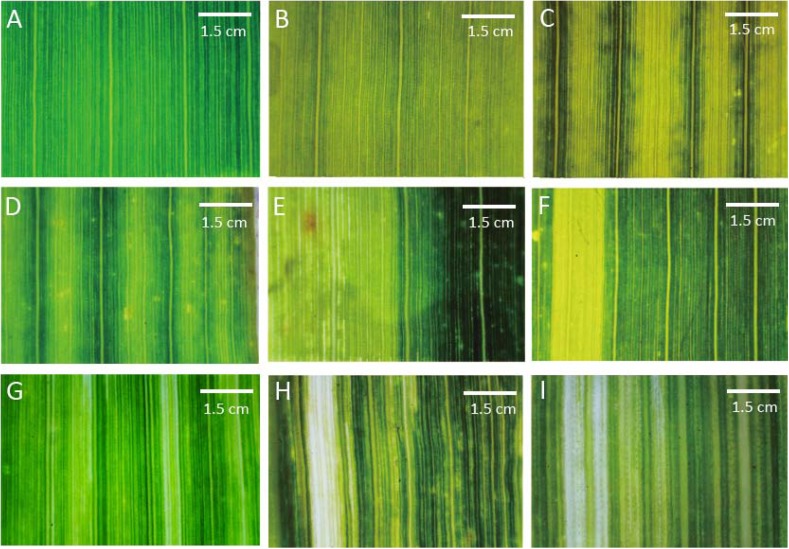
Mutant classifications after a microscopic analysis of leaf vein arrangements. **(A)** Wild-type Yugu1; **(B)** light yellow leaves (*t15*); **(C)** yellow and green striped leaves (*t216*); **(D)** light green striped leaves (*t38*); **(E)** yellow and green leaves (*t261*); **(F)** thick yellow striped leaves (*t267*); **(G)** thin yellow striped leaves (*t198*); **(H)** thick white striped leaves (*t197*); and **(I)** thin white striped leaves (*t189*). Scale: 1.5 cm.

To further characterize leaf vein tissue structures in striped mutants were observed and photographed using a microscope. The wild-type Yugu1 leaves were dark green with uniform leaf veination (Figure 2A). Varying leaf colors were observed among the striped mutants. Most yellow striped mutants produced abnormally colored leaves with a normal leaf veination, including some mutants with fully yellow leaves (e.g., *t15*; Figure 2B). We also detected yellow and green striped mutants with thick light green veins (e.g., *t216*; Figure 2C) as well as light green striped mutants with thick dark green veins (e.g., *t38*; Figure 2D). Yellow and green striped mutants produced yellow as well as green leaves (e.g., *t261*; Figure 2E). Yellow wide striped mutants had leaves that were no longer green (e.g., *t267*; Figure 2F). The presence of striped on these mutants might affect chlorophyll synthesis or degradation or may be related to chloroplast structural damages.

A macro camera was used to observe a few striped mutants, including those with leaf veins that were no longer green and those with irregularly arranged leaf veins. The leaf veins of several yellow narrow striped mutants could not be clearly distinguished (e.g., *t198*; Figure 2G), while an increased number of irregularly arrayed and thick veins were observed in some wide white striped mutants (e.g., *t197*; Figure 2H). Additionally, leaf veins that could not be clearly distinguished were observed in narrow white striped mutants (e.g., *t189*; Figure 2I). The striped of these leaf mutants that were visible to the naked eye and the unclear veins observed by microscopy might be related to the abnormal development of cell structures. Moreover, this could also be the result of plastid mutations (similar to iojap and japonica of maize) or may be due to epigenetic effects of the mutagenesis. This possibility should be the main research focus of future studies involving the screening of leaf vein mutants.

### Results of Iodine Staining

**FIGURE 3:**
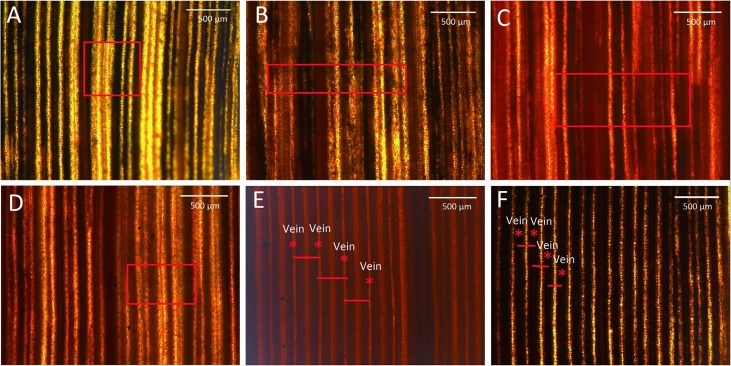
Leaves stained with I_2_-KI. **(A)** Decreased carbohydrate abundance and normal vein arrangement (*t62*); **(B)** decreased carbohydrate abundance and abnormal vein arrangement (*t95*); **(C)** increased carbohydrate abundance (*t66*); **(D)** unevenly stained (*t74*); **(E)** rice (C_3_ control plant); and **(F)** wild-type Yugu1. Scale: 500 μm. Leaf veins are marked with an asterisk, and short red lines represent the distance between adjacent leaf veins. The red boxes in the picture means obvious differ between mutants and wild-type Yugu1.

To more clearly observe the changes in leaf vein structure, the mature leaves from 549 plants were stained with iodine. The distance between leaf veins was greater for the Nipponbare C_3_ control plant than for the Yugu1 C_4_ plant. The average distances between leaf veins were 259.42 μm and 119.21 μm for Nipponbare (Figure 3E) and Yugu1 (Figure 3F) plants, respectively. After iodine staining, carbohydrates appeared bronze-colored and proteins were yellow. In C_3_ plants, photosynthesis occurs in mesophyll cells that are rich in chloroplasts, resulting in the accumulation of carbohydrates. Bundle sheath cells have poorly developed chloroplasts (Langdale, 2011). After iodine staining, the area surrounding the leaf vein was yellow, while the mesophyll cells between leaf veins were brown. The yellow area between leaf veins consisted of bulliform cells with no chloroplasts. In C_4_ plants, photosynthetic carbon fixation mainly takes place in bundle sheath cells, in which many carbohydrates accumulate. Thus, leaf veins were bronze-colored, while the mesophyll cells between the leaf veins were yellow (Figures 3E,F). Iodine staining reflected the accumulation of carbohydrates in leaves and enabled analyses of tissue structure changes due to mutations.

The iodine staining results revealed 52 mutants (Supplementary Figures S1, S2) with four types of abnormal leaf veins, based on the change of accumulation of carbohydrates revealed by color and veins variations detected in mutants (Table 2). We detected 34 Type-A mutants, which had relatively low sugar levels, resulting in an increase in the yellow-stained areas around leaf veins. Additionally, their leaf veins could be clearly distinguished (e.g., *t62*; Figure 3A). Type B included six mutants with relatively low sugar levels and an increase in the yellow-stained areas around leaf veins; however, some leaf veins could not be clearly distinguished (e.g., *t95*; Figure 3B). We identified nine Type-C mutants, with increased sugar levels that led to red-stained areas. These mutants also had irregularly arrayed leaf veins (e.g., *t66*; Figure 3C). Three mutants were categorized as Type D because of the observed uneven distribution of sugars, with increased and decreased sugar levels surrounding leaf veins (e.g., *t49*, *t50*, and *t74*; Figure 3D). Thus, although almost all striped mutants exhibited abnormal sugar accumulation and irregular leaf vein arrangements, we did not detect any mutants with severely defective leaf vein development. This result might be associated with chloroplast development in bundle sheath cells and the inhibition of part of the sugar synthesis and transportation mechanism of a photosynthetic pathway.

**Table 2:** Classification of I_2_-KI–stained mutants based on the change of accumulation of carbohydrates revealed by color and veins variations.

Types	Mutant numbers	Total number	Ratio
A	*t6, t10, t14, t17, t18, t20, t23, t26, t27, t28, t39, t40, t41, t43, t45, t48, t53, t54, t56, t62, t63, t64, t65, t68, t69, t71, t72, t75, t76, t78, t81, t92, t93, t115*	34	65.4%
B	*t11, t46, t47, t95, t104, t106*	6	11.5%
C	*t22, t42, t44, t66, t67, t87, t91, t105, t114*	9	17.3%
D	*t49, t50, t74*	3	5.8%

### Analysis of Resin Sections

**FIGURE 4:**
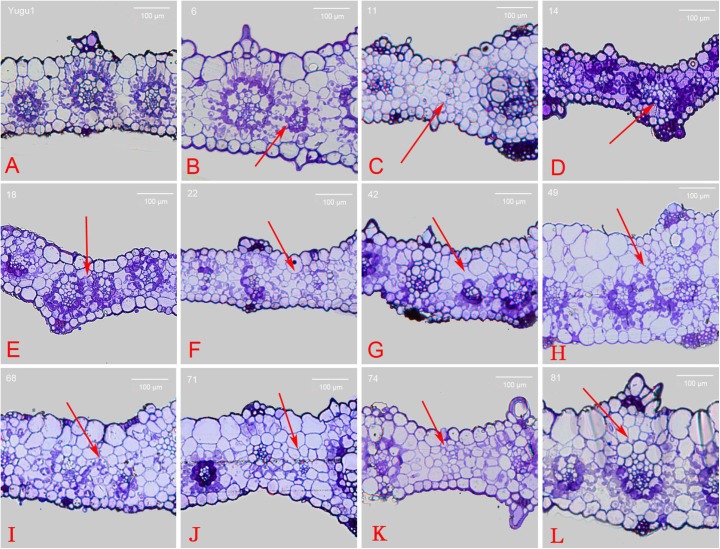
Cross section of wild-type and mutant leaves embedded in resin. **(A)** wild-type Yugu1; **(B)** *t6*; **(C)** *t11*; **(D)** *t14*; **(E)** *t18*; **(F)** *t22*; **(G)** *t42*; **(H)** *t49*; **(I)** *t68*; **(J)** *t71*; **(K)** *t74*; and **(L)** *t81*. Scale: 100 μm. The red arrows indicated the altered part in the leaf tissue cross sections.

According to previously published results (Sage et al., 2014), C_4_ Kranz anatomy possesses enlarged bundle sheath cells compared with C_3_ species. To analyze the cross sections of leaf vein mutants, paraffin and resin sections were prepared for the 52 iodine-stained mutants. Aligned Kranz structures were observed in the Yugu1 leaf cross sections. Mature vascular bundles were surrounded by a layer of large circular or oval bundle sheath cells and an outer layer of spindly mesophyll cells. We detected two or three mesophyll cell layers between bundle sheath cells of two adjacent Kranz structures. Chloroplasts in bundle sheath cells were bluish violet after staining with toluidine blue (Figure 4A).

Abnormally developed Kranz structures were observed in 14 mutants (e.g., Kranz structure dysplasia and fewer mesophyll cell layers), and the red arrows indicated the altered part in the leaf tissue cross sections (Supplementary Figure S3). In the *t6* mutant, vascular tissue that did not develop into a Kranz structure existed between normally developed Kranz structures. This vascular tissue was surrounded by chloroplast-containing bundle sheath cells that were abnormally small and were not associated with tightly arrayed mesophyll cells (Figure 4B). Meanwhile in the *t11* mutant, the vascular bundles were not surrounded by enlarged bundle sheath cells full of chloroplasts. Additionally, mesophyll cells were circular and similarly sized, and some individual vascular tissues were underdeveloped (Figure 4C). The *t14* and *t18* mutants had apparently differentiated bundle sheath and mesophyll cells with both distinct chloroplast distribution and cell size, but the cells were obviously smaller than those of the wild-type leaves. These mutants also had a relatively thin leaf cross section and closely linked adjacent Kranz structures (Figures 4D,E). The *t49* mutant produced mesophyll cells that were bigger than bundle sheath cells, and the cells lacking chloroplasts were also bigger than normal. Moreover, the leaf cross sections were thicker than the wild-type cross sections (Figure 4H). Furthermore, *t22*, *t42*, *t71*, and *t74* mutants produced abnormal Kranz structures that lacked bundle sheath cells surrounding vascular bundles. Additionally, no chloroplasts were detected (Figures 4F,G,J,K). The cross section of the *t68* mutant leaf revealed a disordered cell arrangement, with circular or oval mesophyll and bundle sheath cells (Figure 4I). The *t81* mutant mesophyll cells contained an increased abundance of chloroplasts, while some photosynthetic cells (bundle sheath and mesophyll cells) at the adaxial side of the Kranz structure lacked chloroplasts (Figure 4L).

The *t50* and *t62* mutants developed small, but otherwise normal, Kranz structures. All Kranz structure cells had differentiated normally except for the abnormally small bundle sheath cells surrounding the vascular bundle. The Kranz structure of the *t50* mutant was surrounded by relatively small bundle sheath cells, with an outer layer of large mesophyll cells. In contrast, all of the Kranz structure cells were smaller than normal in the *t62* mutant. The bundle sheath and mesophyll cells of the *t91* mutant differentiated and developed normally, with an orderly arrayed Kranz structure. However, the leaf cross section indicated the parenchymal cells at the adaxial side were larger than normal, resulting in thicker leaves.

## Discussion

### Genetic Analysis of Kranz Anatomy in the Grasses

The identification of mutants in which key characteristics of C_4_ photosynthesis have been disrupted represents a reliable and efficient method for elucidating the molecular basis for these traits. Rizal et al. (2015) identified two sorghum (C_4_ plant) mutants with relatively low leaf vein densities. A further analysis confirmed that the brassinolide synthesis pathway was blocked, implying this pathway may induce an increase in leaf vein density (Rizal et al., 2015). Comparative genomics and bioinformatics investigations led researchers to identify many transcription factors, *cis*-acting elements, and differentially expressed enzyme-coding genes possibly related to the C_4_ pathway based on transcriptome sequencing data. Several transcription factors have been identified, including SCR, SCL23, and SHR, which may regulate both root endodermal cell fate and leaf bundle sheath determination (Slewinski et al., 2012; Slewinski, 2013). Therefore, the genetic network controlling root endodermal cell fate may be wired very similarly to the leaf bundle sheath developmental program. Although some insight has been gained from the genetic analysis of Kranz anatomy in the grasses, much remains to be discovered. Here we show through the characterization of EMS induced mutations of *S. italica*, that forward genetic screens offer an attractive path to understanding Kranz anatomy.

In this study, we identified several mutants with disorderly arrayed leaf veins and undeveloped or abnormally developed bundle sheath cells. For example, the *t22*, *t42*, *t71*, and *t74* mutants lacked large, chloroplast-filled, and sheath-shaped cells surrounding vascular bundles (Figures 4F,G,J,K), suggesting the mutated genes were associated with bundle sheath cell development, or related to the disruption of developmental regulators like SHR or SCR. Thus, these mutants may be useful materials for comprehensive analyses of bundle sheath cell development (e.g., gene localization and cloning studies). We observed that, in addition to not forming normal bundle sheath cells, the mutants produced thin and irregularly shaped leaves, as well as abnormally arranged epidermal and mesophyll cells (Figures 4F,G,I). These complex mutant phenotypes suggest the regulation of the relevant genes or their functional networks is also complex.

Combining with pictures of mutants’ phenotype in field (data were not shown), mutant *t22* and *t71* identified in study exhibited similar plant height, panicle type and leaf size compared with wild-type Yugu1. Especially for *t22*, no significant change of carbohydrate accumulation and vein space (Supplementary Figure S1) has been observed between the mutant and wild type, which might illustrate that genetic dissection of *t22* would definitely provide us more detailed information of Kranz structure formation in Setaria. Further studies focused on genetic dissections of Kranz structure mutants through MutMap or transcriptome approaches would be helpful for clarifying how Kranz anatomy came into being in the grasses. Although we really have acquired one or two mutants that maybe special for Kranz structure, most of mutants identified in this trial may not directly relate to the Kranz structure, and exhibited pleiotropic effect (photosynthetic, chloroplast, leaf size) that may be of limited use for dissecting Kranz development. Furthermore, some Kranz structure mutants may be missed during screening processes, despite this is a fast way for mutant identification. Several severe mutants may not have been identified because Kranz anatomy maybe regulated by many genes and possibly redundant, thus making single or limited gene mutations a difficult way to screen mutations with completely terminated Kranz anatomy. Also, mutations that disrupt Kranz anatomy beyond what was found in this study may be seedling lethal, and thus not detected. Former investigations always only focused on vein density and very less leaf tissue cross sections were identified, this may be why these reports have failed to identify Kranz structure mutants. Large scale of cross section analysis may be the only efficient way to identify Kranz structure variations occurred in mutant library.

### Forward Genetics Analyses of Mutations May Accelerate the Molecular Characterization of the Development of Key Structures in C_4_ Plants

Leaf vein density, bundle sheath cell size, and organelle quantity and localization in bundle sheath cells are key structural features that differ between C_3_ and C_4_ plants. Many researchers are interested in elucidating the mechanisms responsible for the development of these structures. Slewinski have suggested that identifying the positive/negative regulatory factors influencing endoderm development might provide useful information regarding the production of the Kranz structure in C_3_ plants (Slewinski, 2013). Because of a lack of C_4_ mutants with key phenotypes, transcriptomics data have been used to compare whole-genome expression levels between C_3_ and C_4_ plants and to analyze the leaves or specific tissues of C_4_ plants at different developmental stages. By applying genomics and bioinformatics methods, researchers have also identified several transcription factors, *cis*-elements, and differentially expressed genes encoding enzymes possibly related to the C_4_ pathway based on transcriptome sequencing data (Huang and Brutnell, 2016; Huang et al., 2016).

The mechanism responsible for the development of the Kranz structure has not been characterized. In this study, relevant EMS mutants were screened, and the genes associated with these mutants will be cloned. Our data would be combined with the existing gene expression profiles for a subsequent analysis to identify genetic factors that mediate the development of the Kranz structure. Additionally, cloning the related target genes for functional verifications may provide new insights into the evolution and development of the Kranz structure in C_4_ plants.

### Impacts of Chloroplast and Mitochondrial Development Related Genes on Identification of Real C_4_ Related Leaf Striped Mutants

Researchers have studied several leaf striped mutants of rice and maize, and gene localization observations and functional verifications have indicated that striped mutants are usually the result of abnormal chloroplast or mitochondrial development and are severely affected by environmental conditions (e.g., temperature and light) (Yoo et al., 2009; Li P. et al., 2010). We also revealed that many striped mutant phenotypes are affected by the growth period and tissues. Some striped mutants exhibit diverse gene expression patterns during different developmental periods. In some mutants, the striped phenotype might appear only during the seedling stage. For example, the leaves of the *t22* mutant contain white striped during the seedling stage, but then subsequently appear normal. Conversely, the leaves of some mutants, such as *t53*, exhibit obvious striped phenotypes during later growth stages. Other mutants, including *t41*, *t42*, and *t91*, produce a white striped phenotype at all growth and development stages. Leaf abnormalities may also appear at specific sites. For example, the leaf tip of *t19* and *t72* mutants is yellow, while the rest of the leaf is normal, which is in contrast to *t56* leaves, in which white striped are produced toward the bottom.

Leaf veins and Kranz structures developed essentially normally in some striped mutants (e.g., *t56*, *t72*, and *t91*). The Kranz structure is a core structural feature of C_4_ plants. Identifying mutants with significant Kranz structural changes, but that are otherwise normal, is critical for elucidating the development of this structure. Microscopy techniques are invaluable for this type of research. However, an initial analysis of untreated leaf sections may be advisable. Preparing paraffin or resin sections of specific mutants selected based on the initial universal screen of mutants may decrease the workload and improve efficiency.

## Conclusion

In this study, the emerging model species of Setaria was used for dissecting C_4_ Kranz anatomy through forward genetic approaches. Many stable EMS-induced mutants with abnormal Kranz structures in foxtail millet were identified for advancing research efforts on verifying the molecular mechanisms of this important anatomic structure in formation of C_4_ photosynthetic activities. Results of this trial would lay out the foundation of the development of Setaria as an essential model for deciphering genetic basis of plant C_4_ photosynthesis in future.

## Author Contributions

XD designed the experiments. ML, SZ, CT, GJ, and ST performed the experiments. HZ provided materials. ML, SZ, CT, and GJ analyzed the data. ML, GJ, and XD wrote the paper.

## Conflict of Interest Statement

The authors declare that the research was conducted in the absence of any commercial or financial relationships that could be construed as a potential conflict of interest.

## Footnotes

1 https://c4rice.com

2 https://imagej.nih.gov/ij/
