# Supplementary material for: Screening of Mutants Related to the C4 Photosynthetic Kranz Structure in Foxtail Millet
Source: Front Plant Sci. 2018 Nov 14;9:1650. doi: 10.3389/fpls.2018.01650 (PMC6246719; doi:10.3389/fpls.2018.01650)
Supplement: Supplementary file 1 [file Data_Sheet_1.PDF]

Supplementary Material

A Screening of mutants related to the C_4 photosynthetic Kranz structure in foxtail millet

Mingzhao Luo, Shuo Zhang, Chanjuan Tang, Guanqing Jia*, Sha Tang, Hui Zhi, Xianmin Diao *

*** Correspondence:**

Guanqing Jia: jiaguanqing@caas.cn

Xianmin Diao: diaoxianmin@caas.cn

Supplementary Figure

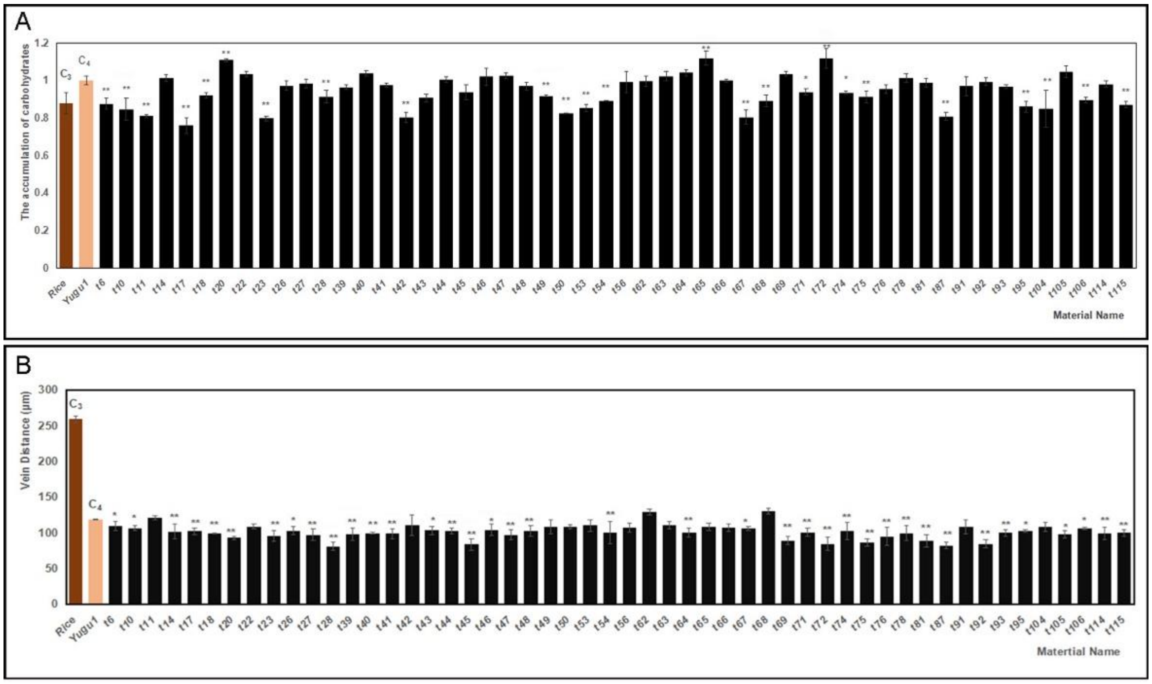

Supplementary Figure 1. The accumulation of carbohydrate (A) and vein distance(B) variations of 52 mutant lines identified by Image J. Comparisons between mutants and wild-type (WT) were determined by Student's t-test. '*' means significance level is below 0.05, and '**' means significance level is below 0.01.

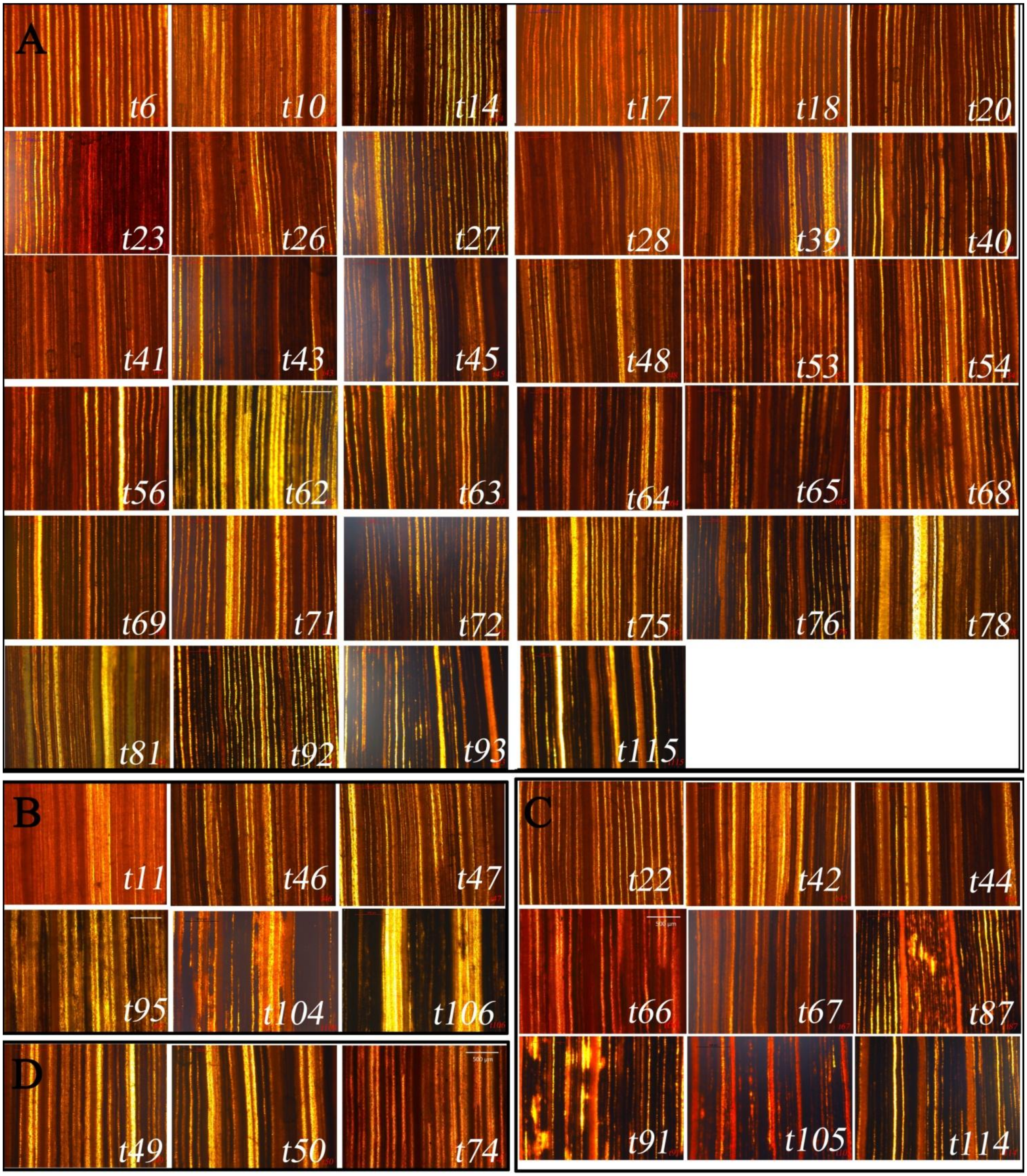

Supplementary Figure 2. Four types(A-D) of abnormal leaf veins, based on the change of accumulation of carbohydrates revealed by color and veins variations detected in mutants after iodine staining.

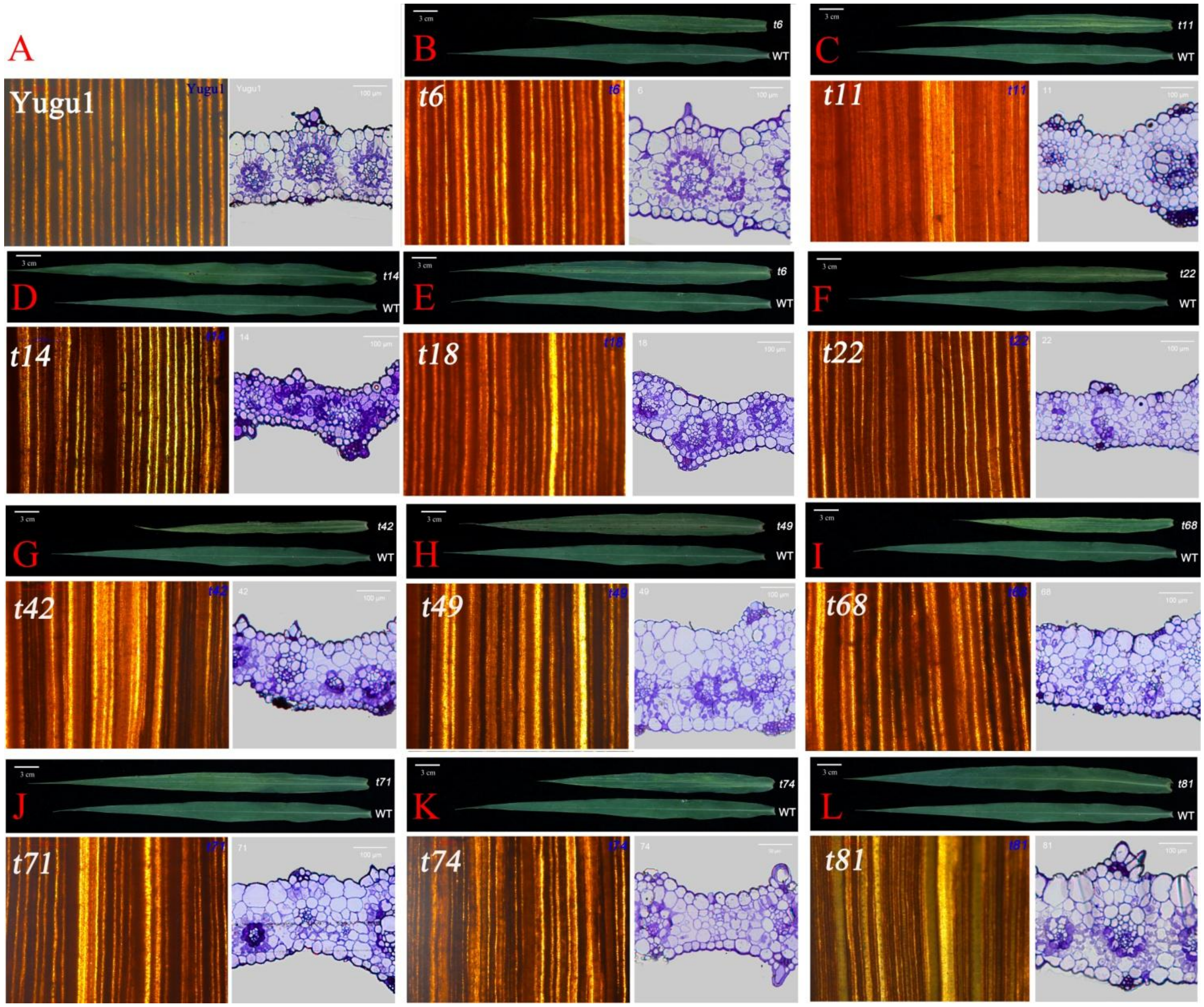

Supplementary Figure 3. Abnormally developed Kranz structures (A-L) were observed.
